# Supplementary figures and images for: Comparison of the Intestinal Structure and Intestinal Microbiome between Two Geographically Isolated Populations of Culter alburnus
Source: Animals (Basel). 2022 Jan 31;12(3):342. doi: 10.3390/ani12030342 (PMC8833785; doi:10.3390/ani12030342)

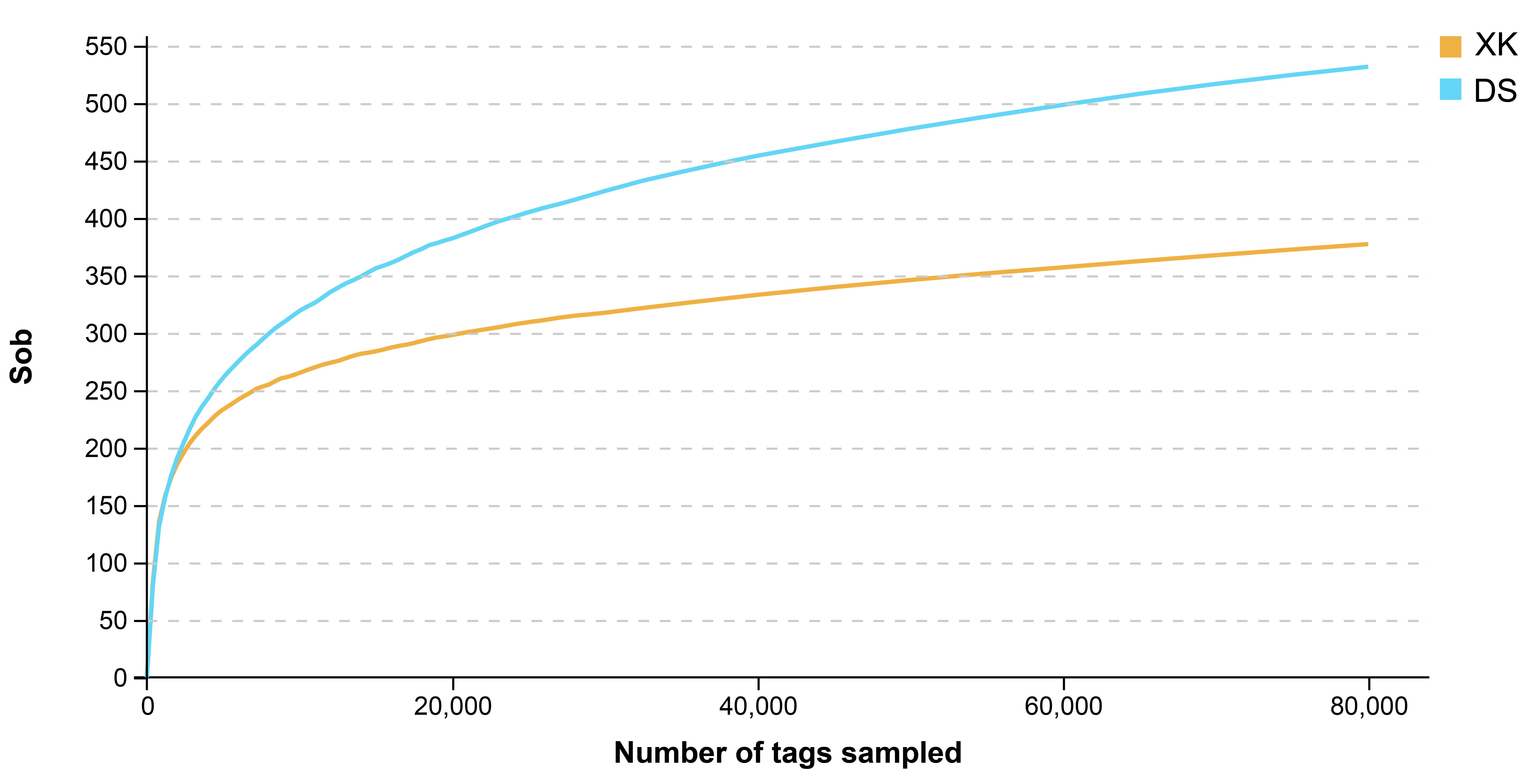

Supplement: Supplementary file 1 [file animals-12-00342-s001.zip › Supplementary Files/Figure S1.jpg]
